# Non-invasive versus invasive management in patients with prior coronary artery bypass surgery with a non-ST segment elevation acute coronary syndrome: study design of the pilot randomised controlled trial and registry (CABG-ACS)

**DOI:** 10.1136/openhrt-2015-000371

**Published:** 2016-04-20

**Authors:** Matthew M Y Lee, Mark C Petrie, Paul Rocchiccioli, Joanne Simpson, Colette Jackson, Ammani Brown, David Corcoran, Kenneth Mangion, Margaret McEntegart, Aadil Shaukat, Alan Rae, Stuart Hood, Eileen Peat, Iain Findlay, Clare Murphy, Alistair Cormack, Nikolay Bukov, Kanarath Balachandran, Richard Papworth, Ian Ford, Andrew Briggs, Colin Berry

**Affiliations:** 1 West of Scotland Heart and Lung Centre, Golden Jubilee National Hospital, Glasgow, UK; 2 Institute of Cardiovascular and Medical Sciences, University of Glasgow, Glasgow, UK; 3 Western Infirmary, Glasgow, UK; 4 Royal Alexandra Hospital, Paisley, UK; 5 Glasgow Royal Infirmary, Glasgow, UK; 6 Royal Blackburn Hospital, Blackburn, UK; 7 Robertson Centre for Biostatistics, University of Glasgow, Glasgow, UK; 8 Department of Health Economics and Health Technology Assessment, University of Glasgow, Glasgow, UK

**Keywords:** Invasive, CABG, NSTE-ACS, RCT, Registry

## Abstract

**Introduction:**

There is an evidence gap about how to best treat patients with prior coronary artery bypass grafts (CABGs) presenting with non-ST segment elevation acute coronary syndromes (NSTE-ACS) because historically, these patients were excluded from pivotal randomised trials. We aim to undertake a pilot trial of routine non-invasive management versus routine invasive management in patients with NSTE-ACS with prior CABG and optimal medical therapy during routine clinical care. Our trial is a proof-of-concept study for feasibility, safety, potential efficacy and health economic modelling. We hypothesise that a routine invasive approach in patients with NSTE-ACS with prior CABG is not superior to a non-invasive approach with optimal medical therapy.

**Methods and analysis:**

60 patients will be enrolled in a randomised clinical trial in 4 hospitals. A screening log will be prospectively completed. Patients not randomised due to lack of eligibility criteria and/or patient or physician preference and who give consent will be included in a registry. We will gather information about screening, enrolment, eligibility, randomisation, patient characteristics and adverse events (including post-discharge). The primary efficacy outcome is the composite of all-cause mortality, rehospitalisation for refractory ischaemia/angina, myocardial infarction and hospitalisation for heart failure. The primary safety outcome is the composite of bleeding, stroke, procedure-related myocardial infarction and worsening renal function. Health status will be assessed using EuroQol 5 Dimensions (EQ-5D) assessed at baseline and 6 monthly intervals, for at least 18 months.

**Trial registration number:**

NCT01895751 (ClinicalTrials.gov).

**Key questions**

**What is already known about this subject?**

- Patients with a prior coronary artery bypass graft (CABG) were excluded from some of the key clinical trials of routine invasive management versus conservative management. There is an evidence gap on the safety and efficacy of invasive management in these patients.

**What does this study add?**

- We will obtain ‘proof-of-concept’ information on feasibility, safety and potential efficacy of a non-invasive approach including optimal medical therapy compared with a routine invasive approach in patients with an acute non-ST segment elevation acute coronary syndrome (NSTE-ACS) with prior CABG, including health outcomes beyond 1 year.

**How might this impact on clinical practice?**

- The results of our trial will provide preliminary information on the rationale for routine non-invasive management in medically stable patients with prior CABG and a NSTE-ACS. The results of this pilot trial will inform the rationale for a substantive clinical trial that would be designed and powered to assess the effects of routine non-invasive versus routine invasive management on health and economic outcomes in this patient group. If our hypothesis proves correct, then routine non-invasive management could be initially adopted for patients except the minority with recurrent ischaemia. Results from a future substantive trial would have the potential to be implemented in routine clinical practice, with the potential to reduce variations in clinical practice, enable more efficient resource utilisation and allow patients with NSTE-ACS with prior CABG to reach critical points in the care pathway more quickly.

## Introduction

Occlusive disease of saphenous vein coronary artery bypass grafts (CABGs) is a common occurrence within 10 years of surgery,^1–3^ meaning patients with prior CABG have a progressive longer term risk of recurrent ischaemia, including angina and myocardial infarction, heart failure (HF) and death. Given the large number of CABG survivors in the UK, and the complexities of their health relating to increasing age and comorbidity, this group of patients represents an increasing challenge to healthcare providers globally.

In this article, we describe the clinical issues relating to the management of patients with a non-ST segment elevation acute coronary syndrome (NSTE-ACS). We then discuss the rationale and study design of a randomised controlled pilot trial of routine invasive management versus conservative non-invasive, and a prospective screening log and registry of patients who were not randomised.

*Epidemiology*: CABG surgery is commonly performed worldwide based on historical evidence of benefit over medical therapy and recent evidence of prognostic benefits over percutaneous coronary intervention (PCI).^4^ ^5^ However, occlusive saphenous vein graft disease affects approximately 1 in 10 patients within 12 months of surgery,^1^ 1 in 5 patients by 3 years^3^ and two-thirds of patients by 10 years.^1^ ^2^ Furthermore, disease in the native coronary arteries may also progress and calcify. Consequently, even though vein graft occlusion may initially be subclinical, angina or myocardial infarction (MI) occurs in most patients by 10 years after CABG surgery. Repeat revascularisation (almost always by PCI) may be required in approximately two-thirds of patients by 12 years,^6^ ^7^ and invasive procedures in patients with prior CABG may be associated with a higher risk of complications acutely and suboptimal outcomes in the longer term.^6^ ^7^

*Scale of the matter*: Chest pain is the commonest reason for hospital admission in the UK and more than 1 in 10–15 patients admitted to hospital with an acute NSTE-ACS have a history of prior CABG.

*(Lack of) clinical evidence, controversy, rationale*: Pivotal clinical trials that compared routine invasive management versus conservative non-invasive management in unstable coronary syndromes, for example, Thrombolysis In Myocardial Infarction IIIb (TIMI IIIb),^8^ Fast Revascularisation during InStability in Coronary artery disease (FRISC II) Value of First Day Angiography/Angioplasty In Evolving Non-ST Segment Elevation Myocardial Infarction: An Open Multicenter Randomised Trial (VINO)^10^ and Randomised Intervention Treatment of Angina 3 (RITA 3),^11^ excluded patients with prior CABG (tables 1 and 2).

**Table 1:** Clinical trials of invasive versus conservative management in patients with NSTE-ACS*

Trials with CABG patients	Year published	Trials without CABG patients	Year published
VANQWISH^22^	1998	TIMI-IIIb^8^	1994
TRUCS^23^	2000	FRISC II^9^	1999
TACTICS-TIMI18^10^ ^24^	2001	RITA-3^11^	2002
ICTUS^25^	2005	VINO^26^	2002
CABG-ACS^27^	After May 2014		
Italian Elderly ACS Study^40^	2012		
OASIS-5 women^41^	2012		

*TACTICS is the only trial to have reported results with specific reference to patients with NSTE-ACS with prior CABG.^24^

CABG-ACS, Coronary Artery Bypass Graft-Acute Coronary Syndrome; ICTUS, Invasive versus Conservative Treatment in Unstable Coronary Syndromes; NSTE-ACS, non-ST segment elevation acute coronary syndromes; TRUCS, Treatment of Refractory Unstable angina in geographically isolated areas without Cardiac Surgery; VANQWISH, Veterans Affairs Non–Q-wave Infarction Strategies In Hospital trial.

**Table 2:** Clinical trials of invasive versus conservative management in patients with a non-ST segment elevation acute coronary syndrome, which included patients with prior CABG surgery

Trials with CABG patients	Year published	Sample size, all participants	Sample size, participants with prior CABG	Prior CABG in invasive group	Prior CABG in conservative group	Minimum duration of follow-up for primary end point, months	Primary and secondary outcomes
VANQWISH^22^	1998	920	156	88	68	12	Death and non-fatal MI
TRUCS^23^	2000	148	18	10	8	12	Death and non-fatal MI
TACTICS-TIMI18^10^ ^24^	2001	2220	484	243	241	6	Death, non-fatal MI, rehospitalisation for acute coronary syndrome
ICTUS^25^	2005	1200	105	62	43	12	Primary end point of death, non-fatal MI or rehospitalisation for anginal symptoms
CABG-ACS^27^	After May 2014	60	60	31	29	18	All-cause mortality, rehospitalisation for refractory ischaemia/angina, MI or heart failure
Total		4548	823 (339)	434 (191)	389 (148)	–	

CABG-ACS, Coronary Artery Bypass Graft-Acute Coronary Syndrome; MI, myocardial infarction.

Clinical guidelines recommend a routine early invasive strategy in higher risk patients with NSTE-ACS.^12–14^ However, invasive management is performed less often in patients with NSTE-ACS with prior CABG, probably because the balance of risks and benefits is less favourable in these patients compared with in NSTE-ACS without prior CABG.^15–17^ Furthermore, when invasive management is performed, PCI is less likely in patients with prior CABG,^16–18^ implying a lower likelihood of benefit with a routine invasive strategy. Real-world evidence implies clinical practice departs from the results of systematic reviews^19^ and the guidelines.^20^ Overall, evidence is lacking to inform the validity of current guideline recommendations,^12–14^ and in our view, the safety and effectiveness of a routine invasive approach in patients with prior CABG and NSTE-ACS is called into question.

### Non-invasive diagnostic testing

CT coronary angiography is useful for imaging graft patency; however, it has limited utility to resolve stenosis severity in chronic calcific coronary disease. Routine stress testing in patients with recent NSTE-ACS and known coronary disease is not evidence-based. Stress tests are used variably in clinical practice (or not at all), according to clinician preference, patient eligibility and local availability. Stress perfusion cardiac MRI (CMR) might be diagnostically useful; however, it is impractical, since a routine strategy based on CMR would be qualified because of lack of availability (eg, stress CMR is not uniformly available across the National Health Service (NHS), especially for patients in an urgent care pathway), patient ineligibility (eg, pacemaker) or non-compliance (eg, claustrophobia, back pain).

### Advances in interventional management

PCI continues to evolve through advances in technologies and technical skills. In recent years, specialist techniques have developed with ‘antegrade’ and ‘retrograde’ approaches to recannalise chronic totally occluded coronary arteries.^21^ The potential benefits of new approaches for complex PCI in CABG patients merit prospective evaluation in the future trial.

*Overall hypothesis*: Considering efficacy, safety and health economics, a routine conservative (and selectively invasive) approach in patients with NSTE-ACS with prior CABG will be an acceptable alternative to the routine invasive approach, which is the current standard of care.

*Active efficacy hypothesis*: Compared with a routine non-invasive approach, a routine invasive approach in patients with NSTE-ACS with prior CABG is associated with a lower rate of all-cause mortality, recurrent MI or HF.

*Active safety hypothesis*: Compared with a routine invasive approach, a routine non-invasive approach in patients with NSTE-ACS with prior CABG is associated with a lower rate of adverse events related to safety during the index hospitalisation.

*Health economics hypothesis*: Compared with a routine invasive approach, a routine non-invasive approach is cost-saving and associated with reduced resource utilisation.

*Pilot study objectives to inform a potential future clinical trial*: To obtain information on: (1) the absolute number of patients admitted with an NSTE-ACS and a prior history of CABG; (2) number of screen failures (and the reasons); (3) enrolment rate; (4) randomisation rate; (5) reasons for failure to obtain informed consent in potentially eligible patients or failure to randomise (clinical registry); (6) medical profile, including comorbidity; (7) revascularisation rates with PCI or CABG; (8) duration of index admission; (9) serious adverse events, including rehospitalisation (and the causes), and death (and the causes), as adjudicated by a Clinical Event Committee (CEC) blinded to treatment group assignment. Some pivotal clinical trials excluded patients with prior CABG, which we think qualifies the current guidelines that recommend invasive management in NSTE-ACS and prior coronary disease (including prior CABG).^12^ ^13^

## Methods and analysis/design

**Figure 1:**
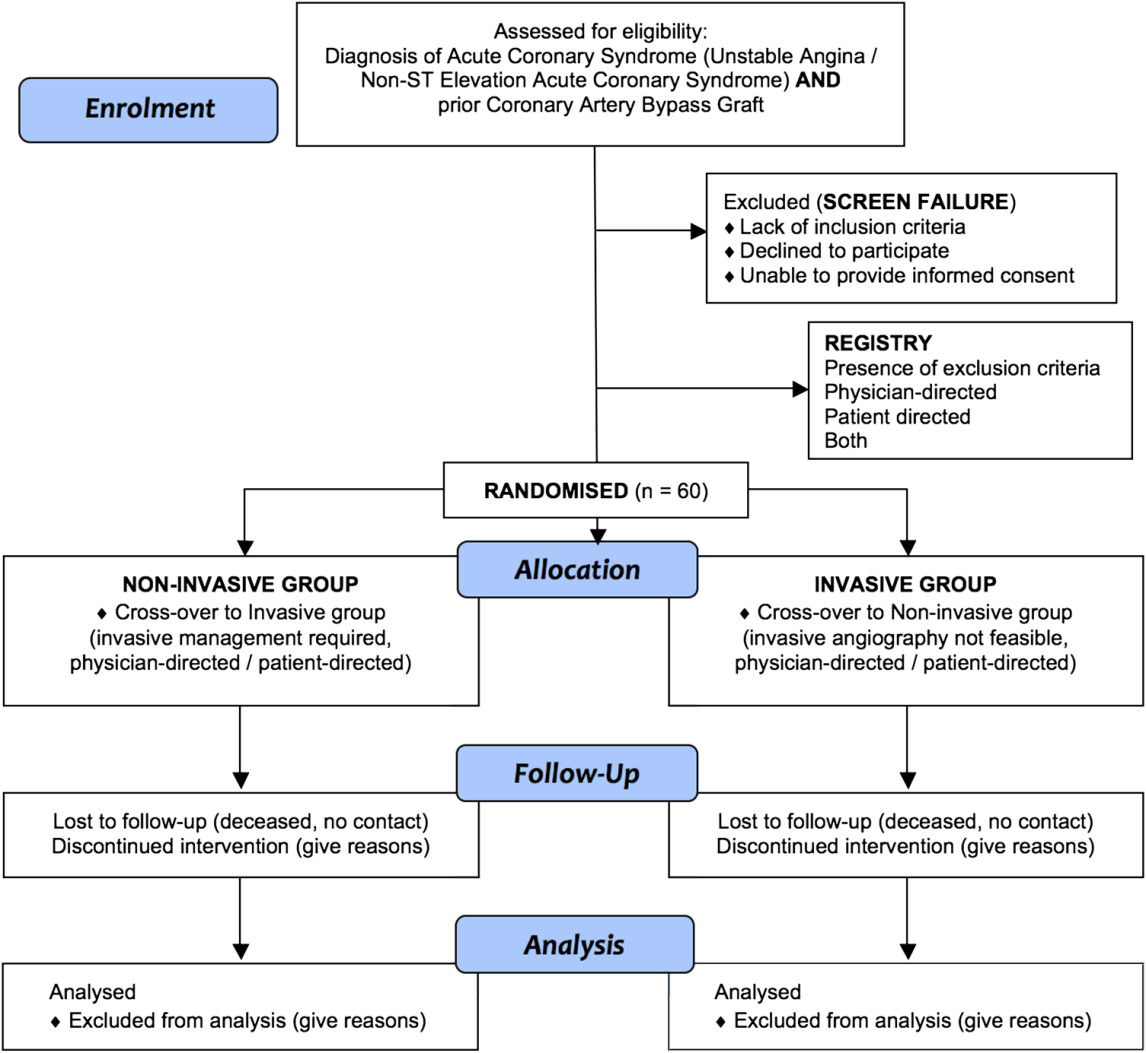
Flow diagram of Coronary Artery Bypass Graft-Acute Coronary Syndrome (CABG-ACS) trial. Patients who provide consent will enter either the (1) randomised trial or (2) registry (ie, based on the presence of exclusion criteria, or physician or patient preference). Patients who do not provide consent and/or are ineligible will enter the (3) screen failure group. A change in the treatment strategy (eg, from non-invasive to invasive management, or vice versa) within the first 30 days from randomisation is a cross-over.

*Overall aim*: To undertake a pilot trial of routine conservative non-invasive management versus routine invasive management in patients with NSTE-ACS with prior CABG who are treated according to standard care with optimal medical therapy (OMT) in NHS hospitals (figure 1). The aim of the pilot is to assess the rationale and provide a rehearsal for the main trial.

*Primary aims*: (1) Compared with routine non-invasive management, to assess if a routine invasive strategy is more effective in symptomatic patients with prior CABG compared with OMT; (2) compared with routine invasive management, to assess if a routine non-invasive strategy is associated with fewer major complications and hence is safer; (3) to prospectively quantify resource utilisation in the NHS with each treatment strategy, derive the difference in the quality-adjusted life years (QALYs) and hence provide preliminary health economic information.

*Secondary aims*: To evaluate: (1) the components of the primary composite outcomes for efficacy and safety; (2) quality of life (EuroQol 5 Dimensions (EQ-5D) at baseline and six monthly intervals for a minimum of 18 months); (3) Canadian Cardiovascular Society (CCS) angina class at baseline and six monthly intervals for a minimum of 18 months; (4) repeat invasive management during follow-up; (5) freedom from coronary and/or bypass graft intervention; (6) secondary care costs; (7) the nature and extent of multimorbidity during the index hospitalisation; (8) the nature and extent of native coronary artery and graft disease, including culprit lesion characteristics; (9) a registry of the patients who were not randomised, including the reasons for not being randomised (physician-directed, patient-directed or both) and their clinical characteristics at enrolment and subsequent outcomes during follow-up.

Accordingly, all invasive coronary interventional procedures will be reported:

- Elective;
- Non-elective unplanned (as adjudicated by the CEC).

*Health economics*: Quality of life, direct healthcare costs, adverse events and their economic consequences over a patient's lifetime are key considerations to inform whether or not a routine conservative approach would be acceptable in patients with NSTE-ACS with prior CABG.^12^ ^28–35^ At this stage, we are uncertain of the potential longer term economic value of routine conservative management. A preliminary economic model will be designed to provide an estimate of the lifetime incremental costs, effects and net monetary benefit of a conservative treatment approach in patients with CABG-ACS. The model will synthesise future data from the pilot trial and the literature (eg, TACTICS CABG analysis).^24^

Second, we are also uncertain on the optimal design of the future trial, including whether health economic results should be the primary objective. *Value of information* analyses will illustrate where additional information in a future trial will have the most value in increasing confidence in net monetary benefit. Such information will provide evidence for the optimal design of a future trial, including considerations of, but not limited to: whether it is needed, sample size, length of follow-up and relevant economic outcomes.

*Pilot*: We aim to prospectively gather information on the overall feasibility of conducting a clinical trial of routine non-invasive versus routine invasive management in recently hospitalised patients with an acute NSTE-ACS and prior CABG. The aim of the pilot is to mimic the main trial and assess whether or not random allocation of these treatment strategies and compliance with the protocol is feasible. Clinical research nurses will support enrolment and follow-up assessments on two of the four sites. NHS clinicians will support enrolment and follow-up assessments on all of the sites. We specifically aim to gather information on screening, recruitment, randomisation (to medical therapy or invasive management), cross-over rates and serious adverse events in patients with prior CABG and a recent NSTE-ACS to inform the feasibility and design of a future definitive clinical trial. Researchers and nurses independent of the clinical team will conduct follow-up for adverse events.

*Consent*: All randomised and registry patients will provide written informed consent as soon as feasible after hospital admission and prior to referral for coronary angiography. Patients will be given an information sheet prior, and may opt out at any time.

## How the sample will be selected: study population

*Setting and feasibility*: The pilot study will involve 60 randomised patients recruited in two large urban hospitals (Glasgow Western Infirmary and Glasgow Royal Infirmary) and two regional district general hospitals (Royal Alexandra Hospital, Paisley and Royal Blackburn Hospital (RBH)). These hospitals were selected in order to reflect the diversity of secondary care in the UK NHS and different models of service provision. One of these hospitals (RBH) has an onsite cardiac catheterisation laboratory, whereas the other hospitals do not. In these hospitals, patients are triaged for invasive management by referral and transfer to the regional cardiothoracic centre (Golden Jubilee National Hospital (GJNH)). The GJNH and RBH provide invasive care for the West of Scotland (2.5 million) and East Lancashire (0.5 million), respectively.

*Screening*: The clinical research team on each site will screen for patients admitted during unscheduled emergency care with a suspected acute NSTE-ACS and prior CABG. Screening will take place in the acute medical and cardiology wards during the course of routine healthcare. Patients who are invited to participate should be eligible for either treatment option. Each patient will be given a corresponding site and study number, and entered into a screening log. Patients who do not provide consent will be included in the ‘screen failure’ log. Only de-identified information will be contained in the screening log including age, gender, medical history, date of admission, date of discharge, angiography (yes/no/date). The community health index (CHI) or NHS number will be recorded to enable electronic record linkage. Patients 18 years and older of both sexes, with a history of NSTE-ACS and who are eligible for either invasive management with coronary and graft angiography or non-invasive management will be invited to participate. Patients who fulfil the inclusion criteria and who do not have exclusion criteria (described in box 1) will be enrolled and randomised as appropriate.

Box 1 **Eligibility criteria for participation in the randomised trial**

**Inclusion criteria**

- Unstable angina or non-ST segment elevation myocardial infarction
- Stabilised symptoms without recurrent chest pain or intravenous therapy for 12 h when ambulant
- Prior coronary artery bypass surgery

**Exclusion criteria**

- Refractory ischaemia (ie, recurrent angina with minimal exertion or at rest (ie, Canadian Cardiovascular Society class III or IV) not controlled by medical therapy)
- Cardiogenic shock
- Inability to give informed consent
- Unsuitable for invasive management

*Randomisation*: After informed consent, patients are randomised with an interactive voice recognition system from the trials unit, to one of two groups: initial medical management or initial invasive management. All patients will receive OMT with treatments prescribed at the discretion of the attending physician, and guidance on uptitration of anti-ischaemic drugs is provided in an investigator guideline. OMT includes dual antiplatelet, antithrombotic and anti-ischaemic therapies as per local protocols and international guidelines.^12^ ^13^

*Non-invasive group*: According to the trial protocol, study participants who have been randomised to the non-invasive group may be referred for invasive management if one of the following prespecified criteria are met: (1) recurrent or refractory (class III or IV) angina with documented ischaemic ECG changes while on ‘optimal’ anti-ischaemic therapy; (2) new ST-segment elevation in two contiguous leads without Q waves or T wave inversion greater than 3 mm or development of haemodynamic instability; or (3) a deterioration in HF status (consistent with Killip class 3 or 4) that the attending clinician judges to be ischaemia-related based on the presence of symptoms, ECG changes and cardiac biomarker elevation.

*Invasive group*: Timed as appropriate according to local hospital protocols. Usually, invasive management is performed early (ie, ≤72 h wherever possible) from hospital admission. Invasive management includes coronary and graft angiography and coronary and/or graft revascularisation with PCI and/or CABG, as clinically appropriate.

*Registry*: Information will be prospectively recorded in a registry for patients with an acute NSTE-ACS and prior CABG who are not randomised. The reasons for non-participation in the randomised trial will be prospectively recorded. The reasons may be the presence of exclusion criteria, unsuitability for either invasive or non-invasive management, physician preference, patient preference, or a combination of these factors. The registry information will provide insights to reflect an ‘all-comers’ real-world population. The registry will also inform on the feasibility of a future substantive randomised trial. The baseline information and the follow-up assessments are intended to be collected in the same way for the registry and randomised trial participants. Information (clinical characteristics, invasive and non-invasive tests, health outcomes and quality of life during follow-up) in the registry patients will be obtained in the same way as the trial patients, that is, with case note review, telephone contact or a hospital visit and during the longer term by electronic record linkage.

*Screen failure*: For screened patients who are (1) eligible but do not consent to participate in the randomised trial or registry, or (2) ineligible, then the reason(s) for not participating will be documented. No further clinical information will be recorded.

*Sample size calculation*: The sample size in the CABG-ACS pilot trial is n=60 patients. We chose this number in order to gain reasonably representative information on the characteristics of patients with NSTE-ACS with prior CABG in contemporary practice to be enrolled in different secondary care settings in order to be representative of the diversity in UK hospitals. We included four hospitals differing in geographic location, availability of catheter laboratory facilities onsite (or not), and hospital type (academic vs regional).

The sample size was selected to enable the feasibility of randomisation, and the reasons for not being randomised were to be prospectively assessed on multiple occasions. As this pilot was an exploratory, proof-of-concept trial, a sample size calculation was not performed. The trial was designed but not powered to assess for between-group differences in the rates of the primary outcome.

*Outcomes*: Serious adverse events during the index admission and follow-up will be evaluated from review of patient records obtained during usual care, and electronic health databases, including the CHI number and NHS number. The occurrence of all of these outcomes will be prospectively entered into an electronic clinical research form.

### Clinical Event Committee

An independent CEC is proposed to review the primary efficacy and safety end points. The CEC will review cases of interest to determine if they meet the criteria defined in this charter. Causality assessments will not be made by the CEC, nor will the committee possess governance authority. The CEC will be blinded where possible regarding all information relating to the randomisation group. The CEC will include four cardiovascular physicians who have expertise in the diagnosis and treatment of cardiovascular disorders and in the medical aspects of clinical trials. The CEC will have a Chairman and coordinator to assist with preparation of de-identified source clinical data, reports and communication with the Trials Unit. The CEC will follow a predetermined adjudication charter.

*Primary outcome*: The primary outcome is the postrandomisation rate of major adverse events (co-primary composite outcome), including one composite outcome for *efficacy* and one composite outcome for *safety*. The comparison between the incidences of each outcome according to treatment group will assess the between-group difference in the proportion of major adverse events in patients allocated to non-invasive conservative management compared with invasive management. The trial is designed to assess the feasibility of recruiting and randomising such a population but is not powered to detect a between-group difference in clinical outcomes.

*Primary efficacy outcome*: Defined as all-cause mortality, rehospitalisation for refractory ischaemia/angina, MI or hospitalisation for HF. The end points will be assessed during the study until the final randomised patient has completed 18 months follow-up.

*Primary safety outcome*: Defined as bleeding (Bleeding Academic Research Consortium (BARC) types 2–4),^36^ stroke, procedure-related MI (type 4a, universal definition), worsening renal function or haemodialysis during the index hospitalisation.

### Definitions of adverse events

*Death*: All-cause, sudden cardiac death, death due to MI, death due to HF, death due to stroke, death due to extra-axial haemorrhage, death due to cardiovascular operation, death due to other cardiovascular cause (eg, infective endocarditis), presumed cardiovascular death (undetermined cause of death), non-cardiovascular death.^37^ ^38^

*Procedure-related MI:* According to the universal definition of MI. A postprocedure ECG will enable diagnosis of Q versus non-Q MI.

*Stroke* is defined as the presence of a new focal neurological deficit thought to be vascular in origin, with signs or symptoms lasting more than 24 h; subdural haemorrhage.

*Major bleeding* is defined according to the BARC criteria.^36^

*Worsening renal function* is defined as deterioration in estimated glomerular filtration rate ≥25% of baseline during the index admission.

*Follow-up and timing of outcome evaluations*: Follow-up (via telephone contact, clinic visits, letter) with completion of quality of life assessments (EQ-5D) will be maintained at six monthly intervals until a minimum of 18 months follow-up has been reached for the final recruited patient. Data will be held for up to 20 years to enable long-term follow-up analyses.

Following randomisation, clinical assessments will involve gathering information from the standard-of-care clinical reviews (end of hospitalisation, 30–42 days and 1 year) and also from clinical contacts recorded in the patients’ medical records. In West of Scotland hospitals, a single system of electronic patient records is used for all hospital attendances and correspondence with primary care.

*Cross-over*: A cross-over between groups is defined as a change of treatment strategy from invasive to non-invasive management, or vice versa. In addition, we predefined cross-over as occurring within 30 days after randomisation.

### Secondary outcomes

1. Quality of life (EQ-5D assessed at baseline and six monthly intervals for a minimum of 18 months);
2. CCS angina class;
3. Hospitalisation for refractory ischaemia and/or angina: refractory ischaemia/refractory angina, defined as recurrent ischaemic symptoms lasting more than 5 min, while on OMT (at least two antianginal treatments) with documented characteristic ECG changes indicative of ischaemia and requiring an additional intervention. An additional intervention is defined as reperfusion therapy for MI, cardiac catheterisation and insertion of intra-aortic balloon pump or revascularisation procedure (PCI or CABG surgery) within 48 h of the onset of this episode. This definition is in line with the Timing of Intervention in Acute Coronary Syndromes (TIMACS) trial;^37^
4. Repeat invasive management during follow-up;
5. Freedom from coronary and/or bypass graft intervention;
6. Health economics: secondary care costs and procedure-related costs (diagnostic tests, PCI, CABG), hospital bed days including intensive care, high dependency unit, general medical.

### Health economics

1. Estimate the cost-effectiveness of routine invasive approach versus conservative management for patients with NSTE-ACS with prior CABG;
2. Identify where additional information in a future trial will have the most value in increasing confidence in net monetary benefit to the NHS.

Secondary care and procedure-related costs (diagnostic tests, PCI, CABG), hospital bed days (including intensive care, high dependency unit, general medical) will be recorded prospectively for index and subsequent hospitalisations. This information will be used to provide preliminary information on health economics.

Early estimates of cost-effectiveness may exhibit a large degree of decision uncertainty. Designing a definitive trial to assess the relative clinical and economic outcomes is challenging given the paucity of evidence. Important considerations to consider are: whether it is needed, sample size, length of follow-up and relevant economic outcomes. Preliminary economic modelling and value of information methods can help identify where additional information in a future trial will have the most value.^32–34^ The following section describes the methods to be used in the economic evaluation. The proposed plan follows guidance published by the National Institute for Health and Care Excellence (NICE).^39^

The economic evaluation will be a cost-utility analysis will full incremental analysis.

### Perspective, time horizon, discount rate

The analysis will be conducted from the perspective of the NHS and Personal Social Services (PSS). Differences in major adverse cardiac events may result in lifetime differences in cost and health effects. Thus, the analysis will use a lifetime time horizon. A 3.5% discount rate will be used for both health and cost effects.

Value of information methods used to identify which outcomes affect the probability of cost-effectiveness and likelihood of NHS adoption under commonly used decision thresholds (£20 000–£30 000/QALY).^32–35^ Value of information analyses will also be used to estimate the optimal sample size of a future definitive trial.

*Statistics*: The Robertson Centre for Biostatistics will act as an independent coordinating centre for data management and conduct statistical analyses. The Centre is registered with a Clinical Trials Unit (National Institute for Health Research (NIHR) Registration number: 16). Summary statistics will be performed with data from the pilot study and registry. The proportions of patients with adverse events will be assessed with a χ^2^ test. Summary analyses will be performed on the screening log data.

### Statistical analysis plan

#### Baseline data

Baseline characteristics of the randomised participants (split by randomised group) and for the registry participants will be summarised using mean (SD for continuous measurement), or median (lower quartile, upper quartile for skewed data) and count per cent for categorical variables. Baseline characteristics for randomised participants and registry participants will be compared using t tests, Mann-Whitney tests and χ^2^ tests (or Fisher’s exact tests) as appropriate.

#### Longer term clinical outcomes

Numbers of events and numbers (%) of patients with events will be summarised. Time to first event of each type will be analysed using Cox proportional hazards regression models, with estimated hazard ratios, 95% CIs and p values using the Wald statistic. Time to event curves will be created using the Kaplan-Meier method.

#### Shorter term safety outcomes

Numbers of events and numbers (%) of patients with events will be summarised. Relative frequency of event types will be compared using Fisher’s exact tests and ORs and 95% CIs for treatment effects estimated using exact logistic regression.

Changes in quality of life over time will be analysed at each time point using analysis of covariance adjusting for baseline and using repeated measures analysis of postintervention data adjusting for baseline. The prespecified health outcomes will be analysed at each time point using ordinal logistic regression adjusted for baseline.

All serious adverse events will be summarised according to system organ class and preferred term.

## Ethics and dissemination

The research study was reviewed and approved by the National Research Ethics Service (Reference 11-WS-0116).

Potential benefits to participants include avoidance of harmful invasive management and avoidance of longer term stent failure. No additional interventions are proposed nor are procedures withdrawn that would be needed on clinical grounds. While the intention-to-treat in each group is either with non-invasive or invasive management, all treatment options remain available according to patient and physician preference, that is, patients initially randomised to medical therapy may undergo invasive management and vice versa.

*Data management and biostatistics*: For confidentiality, patients shall be assigned an identification code at the time of recruitment.

### Trial management

A *trial management group* including the researchers and Local Principal Investigator on each of the four sites coordinated the study's activities on a day-to-day basis.

The NHS Sponsor monitored the trial. Since the trial was a pilot, there was no Independent Data and Safety Monitoring Committee (IDMC).

*The trial is publically registered*:^27^ http://clinicaltrials.gov/ct2/show/NCT01895751.

*Expected value of results*: Since clinical trials usually excluded patients with prior CABG, clinical guidelines are not evidence-based with respect to this group of patients meaning that clinicians lack knowledge, and a clinical decision in favour of invasive management (as per guidelines) is not well informed. Our project addresses this lack of evidence. If a future substantive trial confirms our overall hypothesis, then routine non-invasive management should be adopted generally, except for the minority with refractory angina who would be selectively referred for invasive angiography. The trial results will help reduce unnecessary and expensive diagnostic investigations (ie, stress tests) and provide clarity for decision-making. The results from a future definitive trial could be rapidly implemented in routine NHS practice. This research proposal will provide essential information to inform the design of the future substantive trial, including whether or not it should be performed and if so, its sample size and primary and secondary outcomes.
